# Maternal quality and safety outcomes for Asians and Pacific Islanders in Hawai‘i: an observational study from five years of statewide data

**DOI:** 10.1186/1471-2393-14-298

**Published:** 2014-08-30

**Authors:** Tetine Sentell, Ann Chang, Yongjun Cheng, Jill Miyamura

**Affiliations:** Office of Public Health Studies, University of Hawai‘i at Mānoa, 1960 East-West Road, Biomed T102, Honolulu, HI 96822 USA; Department of OB/GYN, John A. Burns School of Medicine, University of Hawai‘i, 1319 Punahou St, Suite 824, Honolulu, HI 96826 USA; Biostatistics Core, John A. Burns School of Medicine, University of Hawai‘i, 651 Ilalo Street, Biosciences Building, Suite 211, Honolulu, HI 96813 USA; Hawai‘i Health Information Corporation, 733 Bishop St, Honolulu, HI 96813 USA

**Keywords:** Asian, Pacific Islanders, Obstetrics, Health care quality, Cesarean

## Abstract

**Background:**

Empirical evidence regarding maternal quality and safety outcomes across heterogeneous Asian and Pacific Islanders subgroups in the United States is limited, despite the importance of this topic to health disparities research and quality improvement efforts.

**Methods:**

Detailed discharge data from all Hawai‘i childbirth hospitalizations (n = 75,725) from 2008 to 2012 were considered. Validated measures of maternal quality and safety were compared in descriptive and multivariable models across seven racial/ethnic groups: Filipino, Native Hawaiian, other Pacific Islander (e.g., Samoan, Tongan, Micronesian), Japanese, Chinese, white, and other race/ethnicity. Multivariable models adjusted for age group, payer, rural vs. urban hospital location, multiple gestation, and high-risk pregnancy.

**Results:**

Compared to whites, Japanese, Filipinos, and Other Pacific Islanders had significantly higher overall delivery complication rates while Native Hawaiians had significantly lower rates. Native Hawaiians also had significantly lower rates of obstetric trauma in vaginal delivery with and without instruments compared to whites (Rate Ratio (RR):0.66; 95% CI:0.50-0.87 and RR:0.62; 95% CI:0.52-0.74, respectively). Japanese and Chinese had significantly higher rates of obstetric trauma for vaginal deliveries without instruments (RR:1.52; 95% CI:1.27-1.81 and RR:1.95;95% CI:1.53-2.48, respectively) compared to whites, and Chinese also had significantly higher rates of birth trauma in vaginal delivery with instrument (RR 1.42; 95% CI:1.06-1.91). Filipinos and Other Pacific Islanders had significantly higher rates of Cesarean deliveries compared to whites (RR:1.15; 95% CI:1.11-1.20 and RR:1.16; 95% CI:1.10-1.22, respectively). Other Pacific Islanders also had significantly higher rates of vaginal births after Cesarean (VBAC) deliveries compared to whites (RR: 1.28; 95% CI:1.08-1.51) and Japanese had significantly lower rates of uncomplicated VBACs (RR:0.77; 95% CI:0.63-0.94).

**Conclusions:**

Significant variation was seen for Asian and Pacific Islander subgroups across maternal quality and safety outcomes. Notably, high rates of obstetric trauma were seen among Chinese and Japanese vaginal deliveries. Filipinos and other Pacific Islanders had high rates of Cesarean deliveries. Native Hawaiians had better quality and safety outcomes than whites on several quality and safety measures, including obstetric trauma during vaginal delivery. Other Pacific Islanders had high rates of VBACs, while Japanese had lower rates. This information can help guide clinical practice, research, and quality improvement efforts.

## Background

Childbirth is the most common reason women are hospitalized in the United States (U.S.) [1]. Childbirth hospitalizations represent a major expense for public and private insurers with a total cost of over $15 billion a year [1]. Despite their high costs, U.S. maternal and child outcomes are worse than most other developed countries across such critical factors as maternal and infant mortality and low birth weight [2, 3], and are notably unequal by race/ethnicity [4, 5]. For these reasons, pregnancy and childbirth has been identified as a national priority area for health care quality improvement [6]. Health care quality has been defined by the Institute of Medicine (IOM) as “safe, effective, patient-centered, timely, efficient, and equitable” [7]. Safety, the prevention of harm to patients from their interaction with the health care system, has been called “the foundation upon which all other aspects of quality care are built” [8].

Asians and Pacific Islanders are rapidly growing racial/ethnic groups in the U.S. [9] with particularly large populations in some states, such as California and Hawai‘i, and in some communities, including New York City [10]. Birth outcomes are extremely understudied in these populations [11–14]. Many studies that do include Asian and Pacific Islanders often combine heterogeneous subpopulations due to sample size limitations, likely masking important differences [15–17]. The few studies that conduct detailed comparisons of Asian and Pacific Islander subgroups in the U.S. find significant differences in maternal health outcomes across subgroups [13, 14]. For instance, Filipinos have particularly high rates of lacerations [18], Native Hawaiians have notably high rates of preterm delivery [14], and macrosomia is higher in Pacific Islander women [12]. However, detailed data about many important health outcomes, including childbirth quality and safety, are limited.

The Agency for Healthcare Research and Quality (AHRQ) has developed tools to measure obstetric care quality and safety based on administrative health data [19]. These reflect potentially preventable adverse events and are widely used local, nationally, and internationally [20]. These metrics have many advantages, including extensive measurement development and refinement, integrated risk-adjustment, comparability across multiple locations, and national benchmarks [19–21].

Specifically, the AHRQ metrics are grouped into “quality” and “safety” categories. While associated, these are designed to capture distinct health care system concerns [21]. Inpatient Quality Indicators are measures of factors such as volume and utilization designed to help hospitals identify potentially problematic areas of use [21]. The four AHRQ Inpatient Quality Indicators measures focused on obstetric conditions are the Cesarean delivery rate, the primary Cesarean delivery rate, the Vaginal Birth After Cesarean (VBAC) rate, uncomplicated, and the VBAC, all rate. Patient Safety Indicators are designed to “capture adverse events that patients experience as a result of exposure to the health care system” [19], such as complications and adverse events following surgeries and childbirth. The three AHRQ Inpatient Patient Safety Indicators measures focused on obstetric conditions are the obstetric trauma rate from: 1. Cesarean delivery, 2. Vaginal delivery without instrument, and 3. Vaginal delivery with instrument.

Existing studies have not comprehensively considered these measures across Asian American and Pacific Islander subgroups [15]. This information can provide important benchmarks for quality improvement efforts, especially for locations with large populations of heterogeneous Asian and Pacific Islander groups, and can identify areas of priority and urgency [22]. It also provides data to allow for evidence-based clinical discussions of perinatal risk specific to Asian and Pacific Islander subgroups [12].

The study goal was to fill a key research gap by comparing AHRQ maternal quality and safety outcomes across Asian American and Pacific Islander groups in Hawai‘i, specifically across seven racial/ethnic groups: Filipino, Native Hawaiian, other Pacific Islander (e.g., Samoan, Tongan, Micronesian), Japanese, Chinese, other race/ethnicity and white (as comparison). Our hypothesis was that we would see poorer maternal quality and safety outcomes for Native Hawaiians, Filipinos, and other Pacific Islanders compared to whites. This was based on a large literature showing higher risk for Native Hawaiians, Filipinos, and other Pacific Islanders compared to whites across multiple domains associated with poor maternity-related outcomes (e.g., smoking, lower socioeconomic status, higher rates of chronic disease) as well as poorer access to care [23–25].

## Methods

### Data

Detailed discharge data from all Hawai‘i childbirth hospitalizations from 2008 to 2012 were considered using the Hawai‘i Health Information Corporation (HHIC) inpatient all-payer data source [26]. These hospitalizations come from the 15 hospitals in Hawai‘i reporting at least one delivery during this period. Data elements include patient race/ethnicity, age, gender, insurer, length of stay, and diagnosis (based on International Classification of Diseases – 9^th^ revision – Clinical Modification (ICD-9) codes and Medicare Severity-Diagnostic Related Groups (MS-DRGs)). HHIC data links mothers to newborns and includes a unique patient identifier for individuals across all hospitals.

### Sample

We began with the 89,263 vaginal or Cesarean section delivery hospitalizations in Hawai‘i between January 2008-December 2012. Hospitalizations from Tripler, the Department of Defense (DOD) hospital (n = 10,525), were then excluded as this hospital does not consistently report racial/ethnic data. Any other hospitalizations missing race/ethnicity data (n = 3,003) and hospitalizations for payer type as miscellaneous (n = 10) were also excluded. The final sample was 75,725 hospitalizations from 62,316 women.

### Outcome variables

#### AHRQ measures

Using published guidelines [19], we considered four AHRQ maternal-specific inpatient quality indicators. These were the Cesarean delivery rate, the primary Cesarean delivery rate, Vaginal Birth After Cesarean (VBAC) rate, uncomplicated, and VBAC, all. We also considered the three AHRQ maternal-specific inpatient safety measures [15]. These were the obstetric trauma rates from: 1. Cesarean delivery, 2. Vaginal delivery without instrument, and 3. Vaginal delivery with instrument. The specific diagnostic codes used to determine these rates are found in Table 1.

**Table 1:** **MS-DRG and ICD-9-CM codes used to define each maternal health indicator**

	Numerator	Denominator	Exclusion
**Maternal quality indicator**			
Cesarean delivery rate	MS-DRG: 765, 766	MS-DRG: 765, 766, 767, 768, 774, 775	Abnormal presentation, preterm, fetal death, multiple gestation diagnosis codes: ‘64420','64421','65100','65101','65103','65110','65111','65113', '65120','65121','65123','65130','65131','65133','65140','65141', '65143','65150','65151','65153','65160','65161','65163','65180', '65181','65183','65190','65191','65193','65220','65221','65223', '65230','65231','65233','65240','65241','65243','65260','65261', '65263','65640','65641','65643','66050','66051','66053','66230', '66231','66233','66960','66961','67810','67811','67813','7615', 'V271','V272','V273', 'V274','V275','V276','V277'
			Breech procedure codes: '7251', '7252', '7253', '7254'
Primary Cesarean delivery rate	MS-DRG: 765, 766	MS-DRG: 765, 766, 767, 768, 774, 775	Abnormal presentation, preterm, fetal death, multiple gestation diagnoses, or breech procedure(ICD-9-CM codes as the above);
			Previous Cesarean delivery diagnosis codes: '65420','65421','65423'
Vaginal Birth After Cesarean, all rate	MS-DRG: 767, 768, 774, 775	MS-DRG: 765, 766, 767, 768, 774, 775 and diagnosis codes: '65420','65421','65423'	N/A
Vaginal Birth After Cesarean, Uncomplicated rate	MS-DRG: 767, 768, 774, 775	MS-DRG: 765, 766, 767, 768, 774, 775 and diagnosis codes: '65420','65421','65423'	Abnormal presentation, preterm, fetal death, multiple gestation diagnoses, or breech procedure(ICD-9-CM code as the above);
Obstetric Trauma Rate - Vaginal Delivery Without Instrument	MS-DRG: 767, 768, 774, 775 and diagnosis code: '66420', '66421', '66424', '66430', '66431', '66434'	MS-DRG: 767, 768, 774, 775	Instrument-assisted delivery procedure codes: '720', '721', '7221', '7229', '7231', '7239','724','7251','7253','726','7271','7279', '728','729'
Obstetric Trauma Rate - Vaginal Delivery With Instrument	MS-DRG: 767, 768, 774, 775 , diagnosis code: '66420', '66421', '66424', '66430', '66431','66434', and procedure codes: '720','721','7221','7229','7231','7239','724', '7251','7253','726','7271','7279','728','729'	MS-DRG: 767, 768, 774, 775 and procedure codes: '720', '721', '7221', '7229', '7231','7239','724','7251','7253','726','7271', '7279', '728','729'	N/A
Obstetric Trauma Rate - Cesarean delivery	MS-DRG: 765, 766 and diagnosis codes: '66420','66421','66424','66430','66431', '66434'	MS-DRG: 765, 766	N/A
**Non-complicated deliveries**			
Ideal Delivery Rate	Diagnosis code not in (664.2, 664.3 665.5 665.4, 664.4, 664.8, 664.9, 665.8, 665, 665.1, 674.1, 666.0, 666.1, 666.2, 666.3, 665.7, 664.5, 670.xx, 038.xx, 658.4, 659.2, 659.3, 674.2, 674.3, 668.xx, 349.xx 671.4, 673.0, 673.1, 673.2, 673.3, 518.xx (except 03, 06, 83, 84, 89), 998.xx, 669.0, 669.1, 669.2, 669.3, 669.4); Procedure codes not in (6831-6849, 6861-6869, 689, 99.0x)	MS-DRG: 765, 766, 767, 768, 774, 775	N/A

#### Total maternal complications

We also considered a measure of positive maternal outcomes. To do this, we followed the methods of Gregory et al. (2009) who suggested that a useful metric, easily calculated and readily understood by health care consumers, could be based on the proportion of women delivering without maternal or newborn childbirth morbidity [27]. Their metric, which they termed the “ideal delivery (ID) rate”, measures the proportion of positive birth outcomes from total deliveries. We followed their published guidelines [27] to calculate maternal or newborn childbirth morbidity, identified from the ICD-9 codes indicating 26 possible problematic outcomes, including bladder lacerations, pelvic hematoma, neonatal birth trauma, and neonatal respiratory problems. The rates of ideal deliveries (those that did not include the problematic outcomes) were calculated for each Asian and Pacific Islander subgroup. The diagnostic codes specifically used to create these rates are found in Table 1.

### Race/ethnicity

Outcomes were compared in descriptive and multivariable models across seven racial/ethnic groups: Filipino, Native Hawaiian, other Pacific Islander (e.g., Samoan, Tongan, Micronesian), Japanese, Chinese, white, and other race/ethnicity using HHIC race/ethnicity classifications. The race/ethnicity variable is created from the race/ethnicity categories available across all hospitals [26]. HHIC performs extensive ongoing quality assurance to confirm that racial/ethnic data are uniformly collected across all Hawai‘i hospitals. Only one primary race is reported by hospitals from patient self-report at intake, so mixed race is not available. Mixed-race individuals are represented by self-report of their primary race of identification or were included in the “other” racial/ethnic category if patients did not wish to choose one primary racial/ethnic identification.

### Control variables

Based on previous research [4, 5, 11–14], we included the following variables in our multivariable models to control for their impact on health outcomes by race/ethnicity: age (<20; 20-29; 30-30; 40+), payer (Department of Defense, private, self-pay or public), high-risk (yes or no), and location of the hospital (urban vs. rural). Public insurance included Medicare and Medicaid. High risk was measured using previous research [27] and included measures of factors such as chronic disease and substance abuse. Location of the hospital (urban vs. rural) was included due to the expectations of more limited resources and distinct case mixes in rural hospitals. Multiple gestation was also included as a control variable when relevant. (Following the AHRQ metrics [19], births for multiple gestation are excluded from the measurement of Cesarean delivery rate, primary Cesarean delivery rate, and Vaginal Birth After Cesarean (VBAC) rate, uncomplicated.) We also included newborn birth weight in three categories: <2500 grams (low birth weight), between 2500-4000 grams (normal birth weight), and >4000 grams (high birth weight) in control models.

### Statistical methods

We first summarized outcomes and control variables in descriptive statistics by race/ethnicity using Chi-squared tests or Fisher’s exact tests (for categorical variables) and analysis of variance (ANOVA) or non-parametric Kruskal-Wallis tests, if the normality assumption was not satisfied (for continuous variables). Multivariable generalized linear models using generalized estimating equations (GEE) with binomial distribution, log link function and autoregressive of first order (AR(1)) as working correlation matrices were then used to estimate rate ratios (RR) by race/ethnicity with 95% confidence intervals (CI).

Demographic analyses were based on an individual’s first visit. Other analyses were at the level of hospitalization. Deliveries with twins or multiples had only one hospitalization, but individuals could have multiple deliveries during the study period for separate pregnancies. Statistical models accounted for these repeated measures using GEE. All multivariable models adjusted for age group, payer, rural vs. urban hospital location, high-risk pregnancy and newborn birth weight.

Multiple gestation was included when relevant as described above. Two-sided p-value <0.05 was regarded as statistically significant. All analyses were performed in SAS 9.3 (2011; Cary, NC: SAS Institute, Inc). The study was deemed exempt by the University of Hawai‘i Institutional Review Committee. This research adhered to the Strengthening the Reporting of Observational Studies in Epidemiology (STROBE) guidelines for observational studies as outlined on http://www.strobe-statement.org.

## Results

### Demographics

In descriptive findings (Table 2), significant differences were seen across racial/ethnic groups for all demographic factors (p < .0001 for all demographic comparisons). Native Hawaiians had the highest percentage of births for those under 20 years old (15.9%) and Chinese had the lowest (1.4%). Japanese has the highest percentage of births for those 40 years and older (8.3%), while Native Hawaiians had the lowest (2.5%).

**Table 2:** **Demographics of childbirth hospitalizations in Hawai‘i (2008-2012) from Hawai‘i Health Information Corporation data**

	Chinese	Filipino	Native Hawaiian	Japanese	Other	Other Pacific Islander	White	Total	P-values ^*^
**Childbirth Hospitalizations**	2,335	14,965	17,081	7,888	10,126	8,326	15,004	75,725	
**Patients**	1,968	12,545	13,094	6,608	8,904	6,366	12,831	62,316	
**Multiple maternal hospitalizations during study period (% from total # of maternity-related hospitalizations)**	686 (29.4%)	4,577 (30.6%)	7,145 (41.8%)	2,408 (30.5%)	2,969 (29.3%)	3,570 (42.9%)	4,224 (28.2%)	25,579 (33.8%)	<.0001
**# patients with Multiple maternal hospitalizations (% from # of patients)**	353 (17.6%)	2,252 (17.8%)	3,524 (26.2%)	1,227 (18.3%)	1,120 (13.5%)	1,665 (25.9%)	2,029 (15.8%)	12,170 (19.5%)	<.0001
	**N (%)**	**N (%)**	**N (%)**	**N (%)**	**N (%)**	**N (%)**	**N (%)**	**N (%)**	**P-values** ^*^
**Age**									<.0001
<20	28 (1.4%)	1,029 (8.2%)	2,077 (15.9%)	207 (3.1%)	716 (8.0%)	695 (10.9%)	747 (5.8%)	5,499 (8.8%)	<.0001
20-29	620 (31.5%)	5,971 (47.6%)	7,185 (54.9%)	1,887 (28.6%)	4,189 (47.0%)	3,672 (57.7%)	5,986 (46.7%)	29,510 (47.4%)	<.0001
30-39	1,176 (59.8%)	4,977 (39.7%)	3,510 (26.8%)	3,963 (60.0%)	3,667 (41.2%)	1,803 (28.3%)	5,452 (42.5%)	24,548 (39.4%)	<.0001
40+	144 (7.3%)	568 (4.5%)	322 (2.5%)	551 (8.3%)	332 (3.7%)	196 (3.1%)	646 (5.0%)	2,759 (4.4%)	<.0001
**Payer**									<.0001
Dept of Defense	18 (0.9%)	216 (1.7%)	118 (0.9%)	72 (1.1%)	419 (4.7%)	75 (1.2%)	1,121 (8.7%)	2,039 (3.3%)	<.0001
Private	1,461 (74.2%)	8,550 (68.2%)	6,277 (47.9%)	5,542 (83.9%)	4,882 (54.8%)	1,861 (29.2%)	7,757 (60.5%)	36,330 (58.3%)	<.0001
Public	451 (22.9%)	3,711 (29.6%)	6,650 (50.8%)	776 (11.7%)	3,175 (35.7%)	4,221 (66.3%)	3,866 (30.1%)	22,850 (36.7%)	<.0001
Self Pay	38 (1.9%)	68 (0.5%)	49 (0.4%)	218 (3.3%)	428 (4.8%)	209 (3.3%)	87 (0.7%)	1,097 (1.8%)	<.0001
**Hospital**									
Urban	1,831 (93.0%)	9,119 (72.7%)	7,737 (59.1%)	5,453 (82.5%)	6,322 (71.0%)	5,329 (83.7%)	7,252 (56.5%)	43,043 (69.1%)	<.0001
**High Risk**									
Yes	706 (35.9%)	4,919 (39.2%)	4,907 (37.5%)	2,418 (36.6%)	3,005 (33.7%)	2,404 (37.8%)	4,558 (35.5%)	22,917 (36.8%)	<.0001
**Multiple Gestation**									
Yes	33 (1.7%)	159 (1.3%)	159 (1.2%)	153 (2.3%)	115 (1.3%)	98 (1.5%)	263 (2.1%)	980 (1.6%)	<.0001
**Newborn birth weight**									<.0001
Low(<2500 g)	155 (6.6%)	1,681 (11.2%)	1,306 (7.6%)	729 (9.2%)	752 (7.4%)	670 (8.0%)	917 (6.1%)	6,210 (8.2%)	<.0001
Normal(2500-4000 g)	2,093 (89.6%)	12,809 (85.6%)	14,486 (84.8%)	6,886 (87.3%)	8,777 (86.7%)	6,862 (82.4%)	12,676 (84.5%)	64,589 (85.3%)	<.0001
High(>4000 g)	87 (3.7%)	475 (3.2%)	1,289 (7.5%)	273 (3.5%)	597 (5.9%)	794 (9.5%)	1,411 (9.4%)	4,926 (6.5%)	<.0001

^*^P-values were based on Chi-Square test for categorical variables; P-values were estimate assuming independence within patients. N (%) for age groups, payer type, urban, high risk and multiple gestation were based on patient level (first visit).

Fifty-eight percent of the sample overall had private insurance, but this varied by racial/ethnic group, with 83.9% of Japanese having private insurance (highest percent) compared to only 29.2% of other Pacific Islanders (lowest percent). Other Pacific Islanders (66.3%) and Native Hawaiians (50.8%) were most likely to have public insurance.

High-risk also varied across racial/ethnic groups with rates highest among Filipinos (39.2%) and lowest in whites (35.5%) and the other racial/ethnic group (33.7%). Multiple gestations differed significantly across race/ethnicity with the highest rates seen in the Japanese (2.3%) and the lowest rates seen in Native Hawaiians (1.2%). Significant differences were seen in birth weight as well, with Filipinos having the highest percentage of low birth weight and other Pacific Islanders having the largest percentage of high birth weight. While percentages varied by racial/ethnic group, a total of19.5% of patients were included multiple times, indicating they had more than one birth during the five-year study period.

### Clinical quality and safety outcomes

Significant differences (p < .001) were seen for all birth outcomes by racial/ethnic subgroup (Table 3) except for obstetric trauma in Cesarean deliveries, which was extremely rare (less than 15 individuals total) and thus excluded from further analyses. Racial/ethnic differences were then compared in multivariable models (Table 4). Most of the significant differences seen by race/ethnicity in the descriptive analyses remained in multivariable models. Findings of interest are described below by Asian American and Pacific Islander subgroup.

**Table 3:** **Descriptive results from childbirth hospitalizations in Hawai‘i (2008-2012) from Hawai‘i Health Information Corporation data**

	Chinese	Filipino	Native Hawaiian	Japanese	Other	Other Pacific Islander	White	Total	P-values ^*^
	N (%)	N (%)	N (%)	N (%)	N (%)	N (%)	N (%)	N (%)	P-values ^*^
**Maternal quality indicator**									
Cesarean delivery rate	414 (19.7%)	3,367 (25.8%)	3,509 (22.9%)	1,497 (21.5%)	2,297 (25.2%)	1,713 (23.4%)	3,293 (24.4%)	16,090 (23.9%)	<.0001
Primary Cesarean delivery rate	226 (12.0%)	1,713 (15.4%)	1,559 (11.9%)	820 (13.2%)	1,217 (15.5%)	877 (14.3%)	1,810 (15.3%)	8,222 (14.1%)	<.0001
Vaginal Birth After Cesarean, all rate	42 (16.4%)	316 (14.2%)	333 (12.8%)	114 (12.9%)	174 (12.4%)	357 (25.7%)	233 (12.1%)	1,569 (14.7%)	<.0001
Vaginal Birth After Cesarean, Uncomplicated rate	41 (17.4%)	310 (15.0%)	323 (13.5%)	108 (13.1%)	166 (12.7%)	349 (27.3%)	230 (12.9%)	1,527 (15.4%)	<.0001
Obstetric Trauma Rate - Vaginal Delivery Without Instrument	92 (5.7%)	276 (2.8%)	198 (1.6%)	242 (4.5%)	222 (3.3%)	151 (2.7%)	286 (2.8%)	1,467 (2.8%)	<.0001
Obstetric Trauma Rate - Vaginal Delivery With Instrument	44 (29.7%)	182 (21.6%)	69 (12.6%)	103 (21.4%)	110 (21.2%)	68 (15.8%)	123 (18.8%)	699 (19.3%)	<.0001
Obstetric Trauma Rate - Cesarean delivery	Too small to report	Too small to report	Too small to report	Too small to report	Too small to report	Too small to report	Too small to report	Too small to report	0.188
**Non-complicated deliveries**									
ID Rate	1,827 (78.2%)	11,671 (78.0%)	14,410 (84.4%)	6,166 (78.2%)	8,148 (80.5%)	6,498 (78.0%)	12,718 (84.8%)	61,438 (81.1%)	<.0001

^*^P-values were based on Chi-Square test or Fisher’s exact tests for categorical variables; Kruskal-Wallis Test for continuous variables; p-values were estimated assuming independence within patients. N (%) for the above outcomes were based on hospitalizations.

**Table 4:** **Full models predicting rate ratios ((RR) [95% CI]) for childbirth hospitalization outcomes in Hawai‘i (2008-2012) from Hawai‘i Health Information Corporation data**

	Cesarean Delivery Rate	Primary Cesarean Delivery Rate	Vaginal Birth After Cesarean Rate, All	Vaginal Birth After Cesarean Rate, Uncomplicated	Obstetric Trauma Rate - Vaginal Delivery Without Instrument	Obstetric Trauma Rate - Vaginal Delivery With Instrument	Non-Complicated Deliveries-ID rate
	RR [95% CI]	RR [95% CI]	RR [95% CI]	RR [95% CI]	RR [95% CI]	RR [95% CI]	RR [95% CI]
**Race/Ethnicity**							
Chinese vs. White	0.96 [0.88, 1.05]	1.00 [0.88, 1.14]	0.79 [0.58, 1.08]	0.78 [0.57, 1.06]	1.95 [1.53, 2.48]	1.42 [1.06, 1.91]	0.97 [0.95, 0.99]
Filipino vs. White	1.15 [1.11, 1.20]	1.11 [1.04, 1.18]	0.97 [0.82, 1.13]	0.91 [0.78, 1.07]	1.04 [0.88, 1.23]	1.07 [0.87, 1.31]	0.96 [0.95, 0.97]
Hawaiian vs. White	1.02 [0.98, 1.06]	0.83 [0.78, 0.88]	0.99 [0.84, 1.17]	0.94 [0.79, 1.11]	0.62 [0.52, 0.74]	0.66 [0.50, 0.87]	1.01 [1.00, 1.02]
Japanese vs. White	0.96 [0.91, 1.02]	1.00 [0.92, 1.08]	0.84 [0.73, 1.03]	0.77 [0.63, 0.94]	1.52 [1.27, 1.81]	1.02 [0.80, 1.29]	0.97 [0.95, 0.98]
Other vs. White	1.14 [1.09, 1.19]	1.16 [1.09, 1.25]	0.87 [0.73, 1.05]	0.82 [0.68, 0.99]	1.21 [1.01, 1.44]	1.06 [0.84, 1.33]	0.98 [0.97, 0.99]
Other PI vs. White	1.16 [1.10, 1.22]	1.16 [1.07, 1.26]	1.28 [1.08, 1.51]	1.23 [1.03, 1.46]	1.09 [0.89, 1.34]	0.80 [0.60, 1.06]	0.97 [0.96, 0.98]
**Age Groups**							
<20 vs. 40+	0.47 [0.44, 0.51]	0.80 [0.72, 0.88]	1.96 [1.39, 2.78]	2.02 [1.41, 2.89]	1.76 [1.26, 2.46]	1.30 [0.85, 1.99]	0.90 [0.88, 0.92]
20-29 vs. 40+	0.60 [0.57, 0.63]	0.66 [0.61, 0.72]	1.55 [1.21, 1.98]	1.50 [1.15, 1.95]	1.32 [0.98, 1.78]	1.41 [0.99, 2.01]	0.96 [0.94, 0.97]
30-39 vs. 40+	0.75 [0.72, 0.79]	0.71 [0.66, 0.77]	1.31 [1.03, 1.67]	1.32 [1.02, 1.71]	1.15 [0.85, 1.55]	1.23 [0.87, 1.74]	0.99 [0.98, 1.01]
**Payer**							
DOD vs. private	0.98 [0.90, 1.07]	0.87 [0.77, 0.99]	0.88 [0.67, 1.14]	0.88 [0.68, 1.14]	0.74 [0.55, 1.00]	0.64 [0.41, 0.98]	1.06 [1.04, 1.08]
Public vs. private	0.98 [0.95, 1.01]	0.84 [0.81, 0.88]	1.23 [1.10, 1.37 ]	1.25 [1.11, 1.41]	0.54 [0.48, 0.61]	0.76 [0.65, 0.90]	1.02 [1.01, 1.03]
Self Pay vs. private	0.84 [0.76, 0.94]	0.82 [0.70, 0.97]	1.28 [0.87, 1.88]	1.23 [0.82, 1.86]	0.90 [0.63, 1.28]	1.09 [0.73, 1.62]	0.98 [0.95, 1.01]
**High Risk**							
Yes vs. No	1.52 [1.48, 1.55]	2.43 [2.25, 2.43]	0.70 [0.63, 0.78]	0.74 [0.67, 0.82]	1.14 [1.03, 1.28]	1.04 [0.90, 1.20]	0.98 [0.97, 0.99]
**Hospital**							
rural vs. urban	1.66 [1.61, 1.71]	1.73 [1.66, 1.81]	0.09 [0.07, 0.12]	0.10 [0.07, 0.13]	0.81 [0.71, 0.92]	0.65 [0.54, 0.77]	1.14 [1.13, 1.14]
**Multiple Gestation**							
Yes vs. No	NA^1^	NA	0.34 [0.15, 0.80]	NA	0.94 [0.47, 1.87]	1.46 [0.82, 2.59]	0.98 [0.97, 1.03]
**Baby Birth Weight**							
Low vs. Normal	1.20 [1.10,1.31]	1.11 [0.97, 1.26]	1.22 [0.90, 1.65]	1.17 [0.87, 1.59]	0.53 [0.42,0.67]	0.75 [0.57,0.99]	0.98 [0.96, 1.01]
High vs. Normal	1.06 [0.97, 1.16]	0.83 [0.73, 0.95]	1.25 [0.92, 1.69]	1.21 [0.89, 1.63]	0.32 [0.25, 0.41]	0.47 [0.33,0.65]	1.04 [1.02, 1.06]

^1^N/A: For cesarean delivery Rate, primary cesarean delivery rate and vaginal birth after cesarean rate, uncomplicated, multiple gestations were excluded out of analyses based on AHRQ quality indicators.

### Chinese

Chinese (5.7%) had the highest percentage of obstetric trauma from vaginal deliveries without instruments. In multivariable models, Chinese women retained significantly higher rates of obstetric trauma for vaginal deliveries without instruments (Rate Ratio (RR): 1.95; 95% CI: 1.53-2.48) compared to whites and Chinese also had significantly higher birth trauma in vaginal delivery with instrument (RR 1.42; 95% CI:1.06, 1.91) in final models. In unadjusted analyses, Chinese also had the lowest unadjusted percentage (19.7%) of Cesarean deliveries. However, this group did not vary significantly from whites for Cesarean deliveries in multivariable models.

### Japanese

In unadjusted analyses, Japanese (4.5%) had the second highest percentage of obstetric trauma from vaginal deliveries. In adjusted models, Japanese (RR: 1.52; 95% CI: 1.27-1.81) had significantly higher rates of obstetric trauma for vaginal deliveries without instruments compared to whites and significantly lower (worse) rates of non-complicated births compared to whites (RR: 0.97; 95% CI: 0.95-0.98). Japanese also had significantly lower rates of VBACs (RR:0.77; 95% CI:0.63, 0.94) compared to whites.

### Native Hawaiians

In unadjusted analyses, Native Hawaiians had the lowest percentage of obstetric trauma from both vaginal deliveries without instruments (1.6%) and vaginal deliveries with instruments (12.6%) across all racial/ethnic groups. In multivariable models, Native Hawaiians had significantly lower rates of obstetric trauma for vaginal deliveries without instruments (RR: 0.62; 95% CI: 0.52-0.74) and with instruments (RR: 0.66; 95%CI: 0.50-0.87) compared to whites. Native Hawaiians also had significantly higher (better) rates of non-complicated births compared to whites (RR: 1.01; 95% CI: 1.00-1.02), the only studied group for which this was the case.

### Filipinos

In unadjusted analyses, Filipinos had the highest percentage Cesarean deliveries (25.8%). In multivariable models, Filipinos had significantly higher rates of Cesarean deliveries compared to whites (RR: 1.15; 95% CI: 1.11-1.20) and significantly lower (worse) rates of non-complicated births compared to whites (RR: 0.96; 95% CI: 0.95-0.97).

### Other Pacific Islanders

In unadjusted analyses, other Pacific Islanders had the highest rates among all the racial/ethnic groups for all vaginal births after Cesarean (VBAC) (25.7%) and uncomplicated VBACs (27.3%). In multivariable models, other Pacific Islanders had significantly higher rates of all VBACs (RR: 1.28; 95% CI: 1.08-1.51) and uncomplicated VBAC (RR: 1.23; 95% CI: 1.03-1.46) compared to whites. Other Pacific Islanders also had significantly higher rates of Cesarean deliveries compared to whites (RR: 1.16; 95% CI: 1.10-1.22). In final models, other Pacific Islanders had significantly lower (worse) rates of non-complicated births compared to whites (RR: 0.97; 95% CI: 0.96-0.98).

## Discussion

Our study hypothesis was that we would see poorer maternal quality and safety outcomes for Native Hawaiians, Filipinos, and other Pacific Islanders compared to whites. Indeed, high rates of Cesarean deliveries were seen among Filipinos and other Pacific Islanders. Also, Filipinos and other Pacific Islanders had significantly higher percentages of complicated births overall compared to whites. These findings reveal poorer outcomes across some maternal quality and safety measures for Filipinos and other Pacific Islanders compared to whites.

However, many maternal quality and safety outcomes were better for Native Hawaiians compared to whites. Native Hawaiians had significantly lower rates of obstetric trauma during vaginal delivery compared to whites and were the only racial/ethnic group studied to have significantly lower percentages of complicated births overall compared to whites. Nor were all maternal quality and safety outcomes worse for other Pacific Islanders. Instead, other Pacific Islanders had VBACs, a desirable outcome [19], at significantly higher rates.

These findings are intriguing. One possible reason could be that the references groups of whites in Hawai‘i may have poorer health outcomes compared to Whites on the continental US. Certainly, the Caucasian population in Hawai‘i is uniquely distributed, including many who have come from the continental US and only remain temporarily for military service, retirement, and employment [23]. Additionally, whites are not typically the healthiest group in Hawai‘i as the health status and life expectancy of the Caucasian population tends to fall below that of the Chinese and Japanese, though typically above that of Native Hawaiians [28]. That said, our findings are supported by previous work in Hawai‘i and elsewhere has found that, despite having higher risk factors for poor outcomes, Native Hawaiians and other Pacific Islander have low rates of some adverse perinatal outcomes (including low birth weight) [11, 29, 30]. This suggests that Native Hawaiians both in Hawai‘i and other locations may have strong protective factors that support positive birth outcomes, such as strong community and family ties [31]. These findings could be related to a literature in the US that has demonstrated low rates of adverse birth outcomes among some disadvantaged (primarily Hispanic) immigrant groups [32].

We also found poor outcomes for some maternal quality and safety measures among Chinese and Japanese women compared to whites. Japanese women had significantly higher percentages of complicated births overall and high rates of obstetric trauma were seen among Chinese and Japanese women with vaginal deliveries. These are interesting findings as Japanese and Chinese populations in Hawai‘i tend to have favorable health profiles, good access to care, and high insurance coverage rates [23, 28]. However, the older populations who show strong longevity may have distinct demographic characteristics compared to those who are young enough to be having children [23], or other risk factors such as English proficiency may be at play [33, 34]. These may also reflect cultural preferences. These are important areas for further study as they suggest distinct intervention and policy solutions.

Our findings support previous research that found high rates of perineal trauma in Asians [12, 14, 35] and add new evidence about this issue. For instance, we find that unadjusted rate of obstetric trauma with an instrument was high among Filipinos in unadjusted analyses (higher than in all other racial/ethnic groups except Chinese). However, the unadjusted rate of obstetric trauma without an instrument was not significantly higher among Filipinos compared to other studied racial/ethnic groups in descriptive analyses.

Little research exists on perinatal outcomes for Pacific Islanders compared to Asian American subgroups in the published literature [12–14]. Previous research has aggregated Native Hawaiian with other Pacific Islander groups [12]. We find notable differences between Native Hawaiians and other Pacific Islanders, particularly in Cesarean delivery rates, which are significantly higher for other Pacific Islanders compared to whites in multivariable models, a difference not seen for Native Hawaiians. As the average weight and height of Pacific Islander women are higher than for many other Asian women [36], this is not surprising. However, this cannot account for the findings of lower primary Cesarean delivery rates in Native Hawaiians. This may be an important area for further study.

Cesarean sections are the most common major surgical procedure in the United States [37]. Promoting appropriate use of Cesarean delivery is a particular focal area for quality improvement [6, 38]. Our research suggests that a better understanding of this topic in Filipinos and other Pacific Islanders may be fruitful toward a goal of Cesarean delivery reduction. The increase in Cesarean section deliveries have been coupled with a decrease in the rate of VBACs [39]. In our study, we see rates of VBACs are very high among other Pacific Islander women.

Our findings of significant variation across Asian and Pacific Islander subgroups for study outcomes have important implications for policy and practice. Because much research combines Asian American and Pacific Islander women together, it can be challenging to understand the specific health risks for subgroup for research and clinical practice [12–14]. Thus, not only does our study highlight the importance of variation, but highlights particular areas for improvement for each racial/ethnic group to potentially improve on these outcomes. This becomes increasing more important as the number of Asian American and Pacific Islander women grow in the U.S.

For example, a recent overview of these metrics using aggregated Asian American and Pacific Islander groups found that “API [Asian and Pacific Islander] women had higher rates of obstetric trauma, both with and without instrument assistance, than other women [15]”. Our study finds considerable differences in this metric for Asian and Pacific Islander subgroups. Chinese (5.7%) and Japanese (4.5%) had the highest percentage of obstetric trauma from vaginal deliveries without instruments while Hawaiians had the lowest percentages (1.6%) in all groups. Filipinos were only high in obstetric trauma with an instrument, but not in obstetric trauma without an instrument compared to other studied racial/ethnic groups. This information suggests different foci for quality improvement efforts for distinct Asian and Pacific Islander groups.

We found higher rates of complicated deliveries in several Asian and Pacific Islander groups compared to whites. Complicated deliveries are considerably more expensive than uncomplicated deliveries [40]. Our findings may be of particular interest to Medicaid, which cared for 37% of the total births and considerably higher percentages of some groups (e.g., other Pacific Islanders) who had poor outcomes. At the same time, obstetric trauma rates were particularly high among Japanese individuals who are primarily insured by private providers in our study. Obstetric trauma can lead to clinical and quality of life issues over time [41] so reducing this risk may be a focus of quality improvement in private providers with large numbers of Japanese members.

### Limitations

This study has several important strengths. We have a full sample with all civilian hospitalizations in a diverse state with detailed race/ethnicity data for five years. Hawai‘i hospitalization data is extremely diverse, including racial/ethnic groups not easily captured in most population-based samples, but that have increasing relevance to the U.S. population generally due to their increasing size [9]. A major strength of the Hawai‘i data is that, because of the state's unique demographic composition, it provides a sufficient sample size to offer a window into the utilization patterns of understudied groups in a timely way while these populations are increasing in other locations. Findings from Hawai‘i can be compared to other settings in the continental US and elsewhere and may provide benchmarks for locations where Asian and Pacific Islander subgroups analyses would not currently be reliable without extremely large samples. Thus, information about maternal health outcomes in Hawai‘i is of relevance to that state, but also provides key comparative data for other locations. Our findings may be particularly informative for states, like California and New York, and for specific communities, such as New York City, San Diego, and Houston, where Asian and/or Pacific Islander populations are particularly high and growing [10].

However, this study also has some limitations. Using administrative data generally and the AHRQ metrics specifically have many advantages as these provide a comprehensive, low burden, low cost method to obtain standardized information on safety events across large populations that incorporate risk and have been developed with extensively clinical and research expertise [21]. These metrics are also readily comparable across multiple locations in the US and even internationally [19–21]. However, administrative data has some potential disadvantages. Our data lacks some relevant demographic information, such as education or health literacy, as well as some relevant clinical data, such as women’s previous birth histories, gestation week, and BMI. These factors could certainly help to explain some of the findings across racial/ethnic groups and may suggest critical areas for interventions [42]. Also, though our models account for public vs. private insurance and maternal age, we are not able to fully adjust for sociodemographic differences between groups. This could be relevant in that Chinese and Japanese women tend to have higher socioeconomic status compared to Native Hawaiian and Pacific Islander women, which may account for some of our findings [23].

At the same time, understanding how patterns of disparities manifest by race/ethnicity is important, even if these patterns can ultimately be explained by other socio-demographic and clinical factors. Additionally, our data provides baseline information about racial/ethnic patterns in a location with large Asian and Pacific Islander populations that could be potentially compared not only to locations with large numbers of these groups, but also in locations that have smaller numbers of these population groups, but who already compile the AHRQ metrics in their state [39, 43].

Also, we only had data on maternal ethnicity and were thus unable to examine the effect that paternal ethnicity may have on perinatal outcomes. We also lack information about maternal birthplace, which may be an issue as foreign-born women may have better outcomes than those born in the US [44]. Additionally, the race/ethnicity variables have limitations. We only report only primary race/ethnicity and our findings may not be generalizable to those of multiple Asian ethnicities [14]. Also, problems in concordance have been found between self-reported race/ethnicity and race/ethnicity recorded in administrative data sets, particularly for minority races [45, 46]. While the HHIC data has strong and consistent protocols in data collection across hospitals in Hawai‘i, it is possible that the racial/ethnic classifications used in this study may not precisely mirror the classifications individuals would choose for themselves. However, the research on this topic concludes that these concordance issues likely serve to underestimate disparities [45]. Thus, better measurement of race/ethnicity would likely only find more of disparities demonstrated here rather than less.

We followed AHRQ guidelines in our metrics, making our research comparable to the extensive existing literature on this topic. However, the AHRQ Cesarean measures have been criticized for excluding many patients with risk factors from the measure instead of risk adjusting for them, which may impact study analyses by race/ethnicity [47]. Also, our outcomes are only indicators of quality, not definitive definitions [48]. Self-reported experiences of health care quality might vary.

## Conclusions

Our study provides a comprehensive portrait of variation in quality and safety outcomes by race/ethnicity across diverse Asian and Pacific Islander racial/ethnic groups. We identify some potential disparities in maternal health outcomes, particularly in obstetric trauma for Chinese and Japanese and Cesarean rates for Filipinos and other Pacific Islanders. This knowledge will help improve the specificity of quality data around maternal outcomes and identify important groups to target for interventions.

Our study also highlights that Asian and Pacific Islanders subgroups must be disaggregated to understand patterns, identify possible disparities, and design effective interventions. This is particularly important to consider in childbirth, which is the most common reason for hospital admission among women. While disparities for Asian or Pacific Islander groups are not uniform, understanding distinct risks and problematic birth outcomes among groups is critical to ensuring health equity among all racial/ethnic groups.

## Abbreviations

AHRQ: Agency for healthcare research and quality
ANOVA: Analysis of variance
API: Asian and pacific islander
CI: Confidence intervals
DOD: Department of defense
GEE: Generalized estimating equations
HHIC: Hawai‘i health information corporation
ICD-9: International classification of diseases – 9^th^ revision – clinical modification codes
ID: “Ideal delivery”
IOM: Institute of medicine
MS-DRGs: Medicare severity-diagnostic related groups
NA: Not applicable
RR: Rate ratio
STROBE: Strengthening the reporting of observational studies in epidemiology
US: United states
VBAC: Vaginal births after cesarean.
